# Association of Higher MERS-CoV Virus Load with Severe Disease and Death, Saudi Arabia, 2014

**DOI:** 10.3201/eid2111.150764

**Published:** 2015-11

**Authors:** Daniel R. Feikin, Basem Alraddadi, Mohammed Qutub, Omaima Shabouni, Aaron Curns, Ikwo K. Oboho, Sara M. Tomczyk, Bernard Wolff, John T. Watson, Tariq A. Madani

**Affiliations:** Centers for Disease Control and Prevention, Atlanta, Georgia, USA (D.R. Feikin, A. Curns, I.K. Oboho, S.M. Tomczyk, B. Wolff, J.T. Watson);; Ministry of Health, Jeddah, Saudi Arabia (B. Al-Raddadi, M. Qutub, O. Shabouni, T.A. Madani);; King Faisal Specialist Hospital and Research Center, Jeddah (B. Al-Raddadi, M. Qutub);; King Abdulaziz University, Jeddah (T.A. Madani)

**Keywords:** Middle East respiratory syndrome coronavirus, MERS-CoV, virus load, Saudi Arabia, viruses, zoonoses

## Abstract

Middle East respiratory syndrome coronavirus (MERS-CoV) causes a spectrum of illness. We evaluated whether cycle threshold (C_t_) values (which are inversely related to virus load) were associated with clinical severity in patients from Saudi Arabia whose nasopharyngeal specimens tested positive for this virus by real-time reverse transcription PCR. Among 102 patients, median C_t_ of 31.0 for the upstream of the E gene target for 41 (40%) patients who died was significantly lower than the median of 33.0 for 61 survivors (p = 0.0087). In multivariable regression analyses, risk factors for death were age >60 years), underlying illness, and decreasing C_t_ for each 1-point decrease in C_t_). Results were similar for a composite severe outcome (death and/or intensive care unit admission). More data are needed to determine whether modulation of virus load by therapeutic agents affects clinical outcomes.

## Abstract 2

More data are needed to determine whether modulation of virus load by therapeutic agents affects clinical outcomes.

Middle East respiratory syndrome coronavirus (MERS-CoV) was first reported in September 2012 in a patient from the Kingdom of Saudi Arabian (KSA) who had pneumonia (*1*). From September 20, 2012 through January 2, 2015, a total of 825 MERS-CoV cases (356 fatal) have been reported from KSA, representing most of the cases worldwide (*2*,*3*). Initial reports of clinical course among MERS-CoV patients from KSA indicated high case-fatality rates (>50%) (*4*,*5*), but the subsequent increase in testing of symptomatic and asymptomatic persons as part of contact investigations has shown that approximately one fifth to one fourth of patients are mildly symptomatic or asymptomatic (*6*,*7*).

The factors dictating severity of illness and outcome among MERS-CoV patients are still not well defined. Underlying illness and older age have been associated with more severe disease and death (*4*,*5*,*8*,*9*). In some other viral respiratory illnesses, the amount of virus measured in the respiratory tract has been associated with more severe disease (*10*–*16*). However, whether virus load of MERS-CoV is associated with severity of illness is unknown. We evaluated this association among a large cohort of MERS-CoV patients from the Jeddah region of KSA during 2014.

## Methods

### Study Population

Eligible persons were children and adults admitted to Jeddah area hospitals during March–May 2014. Patients were included if they met the following criteria: 1) tested positive for MERS-CoV by real-time reverse transcription PCR (RT-PCR) from nasopharyngeal swab, sputum, or bronchoalveolar lavage samples at the Jeddah Regional Laboratory during March 26–May 16, 2014, and 2) were matched to a line list of Jeddah MERS-CoV patients with the most complete clinical and outcome data (*17*)

### Data Collection

Jeddah Regional Laboratory maintained a database of all patients tested that included cycle threshold (C_t_) values for the upstream of the envelope E (upE) gene and open reading frame (ORF) 1a targets. A composite of MERS-CoV patients for whom medical history and clinical information were obtained by case report forms and medical records review or by phone calls was compiled in late May 2014 (*17*). Laboratory and clinical datasets were linked by matching the national identification number for Saudis or foreign identification number for foreigners. If this number was a close but not exact match, confirmatory evidence of identity included sex, age, and date of specimen collection. All personal identifiers, including identification numbers, were stripped from the merged, analytic dataset.

### Specimen Collection and Laboratory Testing

Patients with suspected MERS-CoV infection were tested as soon as possible after admission to Jeddah area hospitals at the discretion of the treating clinicians, under guidance from the KSA Ministry of Health (MOH) (*18*). In addition, some close contacts of confirmed MERS-CoV patients and health care workers who cared for MERS-CoV patients were sampled as part of contact investigations. KSA MOH guidelines recommended collection of nasopharyngeal swab as the screening specimen for all patients suspected to have MERS-CoV but enabled additional collection of lower respiratory tract specimens for intubated patients (*5*,*18*). Swab samples were obtained by using Dacron flocked swabs and placed in 2–3 mL of virus transport media. Specimens were stored at 2°C–8°C and transported to the Jeddah Regional Laboratory within 48 hours after collection.

At the Jeddah Regional Laboratory, total nucleic acid extraction from clinical specimens was performed by using the MagNA Pure LC 96 or the MagNA Pure Compact (Roche, Basel, Switzerland). Both instruments were programmed for the DNA_Blood_100_400_V3_2 protocol (Roche) with a 200-μL sample volume and 100-μL elution volume. Real-time RT-PCR amplification was performed by using the ModularDx Coronavirus SA1 (EMC) upstream E-gene kit (TIB Molbiol LLC, Berlin, Germany) for primary detection, and the ModularDx MERS-Coronavirus (EMC) ORF1a kit (TIB Molbiol LLC) was used to confirm positive results (*5*,*19*). Discordant results needed to be confirmed with a second clinical specimen. C_t_ values were read as positive if the amplification curve crossed the threshold set above the background fluorescence levels. Standard curves were not generated for each real-time RT-PCR run, and absolute virus concentrations in the specimens were not calculated. Because upE and ORF1a C_t_ values were highly correlated (Spearman’s ρ) and because the upE region is noncoding and would be less influenced by the presence of mRNA, all primary analyses were run for upE C_t_ values.

### Outcome and Risk Factor Variables

The main outcome of interest for this study was severity of illness and the main predictor being assessed was the MERS-CoV virus load, as reflected by the C_t_ values. The following severity outcomes were assessed: death by the time of follow-up chart review or phone contact, a composite severe outcome (death and/or admission to the intensive care unit [ICU]), admission to ICU versus admission to the general ward among hospitalized patients, and symptomatic versus asymptomatic infection among surviving patients. Other independent variables in the analysis as risk factors and/or potential confounders of the association between C_t_ value and outcome were underlying illness, age, sex, and week of specimen collection.

C_t_ values were used as a relative indicator of virus load, in that lower C_t_ values were considered to reflect higher virus load than were higher C_t_ values, an intrinsic characteristic of real-time RT-PCR (*12*,*20*). Analysis of the association between C_t_ values and severity was restricted to nasopharyngeal specimens because C_t_ values from lower respiratory tract specimens (e.g., sputum and bronchoalveolar lavage) can be lower (i.e., higher virus load) than upper respiratory tract specimens, thereby introducing a possible bias when evaluating severity (*20*). If multiple specimens were available for a patient, the nasopharyngeal sample with the earliest date of collection was used.

Underlying illness was defined as heart disease, chronic lung disease, renal diseases, and/or diabetes. Age was divided into quartiles based on the age distribution of MERS-CoV–positive patients. To assess whether changes might have occurred over time during the period of testing that confounded the results (e.g., specimen collection or transport methods), we created a categorical variable based on the date of specimen collection for each 2-week period during March 26–May 16. “Days since illness onset” was the difference in the date of the first symptom of MERS (i.e., cough, dyspnea, fever) and the date of specimen collection.

### Statistical Analysis

Because C_t_ distributions were not normally distributed, we compared the C_t_ values between 2 groups using the Wilcoxon rank sum test and for >3 groups using the Kruskal-Wallis test. C_t_ values also were grouped into high virus load (C_t_≤26), medium virus load (C_t_ 27–33), and low virus load (C_t_≥34) values for categorical analysis, and groups were compared by using the χ^2^ test (Brett Whittaker, pers. comm. on grouping of C_t_ values). Correlation between other continuous variables and C_t_ values were compared after the values were ranked and were assessed by Spearman rank correlation coefficient (Spearman’s ρ).

Univariate and multivariate logistic regression was performed to identify risk factors for death, as well as for a composite severe outcome (death and/or ICU admission) among MERS-CoV–positive patients. Categorical variables with missing data were coded to include missing as a valid response category.

Variables with p<0.10 in univariate analysis were included in multivariable analyses, and backward and forward selection methods were performed to identify significant variables at p<0.05 for any category of each variable. The main focus was to determine whether the outcome of interest (i.e., death or composite severe outcome) was more likely as C_t_ values decreased (i.e., virus load increased). A variable was considered to be a significant confounder of the C_t_ association if the parameter estimate for C_t_ changed by >10% and was included in the model regardless of statistical significance. Separate analyses were run for the C_t_ values resulting from the upE and ORF1a RT-PCR targets. All analyses were performed by using SAS 9.3 (SAS Institute, Cary NC, USA). No formal sample size was calculated; rather, all eligible patients during the study period were included in the analysis.

## Results

During the study period, the clinical and laboratory datasets for 120 (50%) of 239 MERS-CoV–positive patients were able to be linked. Seventy-three percent were men, and the median age of all patients was 50 years (range 8–86 years); for 19 patients, information about age was missing. Thirty-two (26%) patients were health care workers, and 65 (56%) were Saudi. Specimens for MERS-CoV were collected in 18 facilities; 59% of specimens were collected from 4 facilities, and the remaining 14 facilities provided <5 samples each. In addition, 15 (13%) specimens were collected at home for asymptomatic or mildly symptomatic contacts.

**Figure 1:**
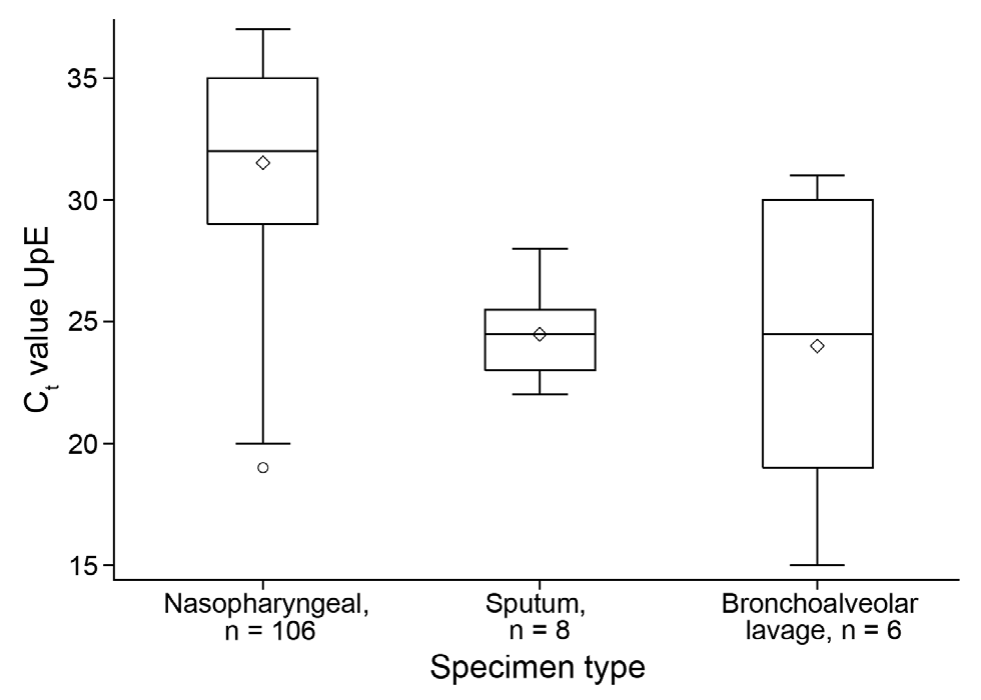
Box plot of C_t_ values for MERS-CoV patients by specimen type, Kingdom of Saudi Arabia, 2014. Box and whiskers plot features are as follows: central line in box is median, bottom line of box is first quartile (25%), top line of box is third quartile (75%), diamond is mean, bottom of whiskers is first quarter minus 1.5 × interquartile range, top of whiskers is third quarter plus 1.5 × interquartile range, and dots are outliers. Groups were compared by using the Kruskal-Wallis test, p<0.0001. C_t_ , cycle threshold; MERS-CoV, Middle East respiratory syndrome coronavirus; upE, upstream of E gene.

Mean and median C_t_ values for the 120 linked MERS-CoV–positive patients for upE were 30.7 and 32.0, respectively (range 15–37). For 118 patients for whom ORF1a results were available, mean and median C_t_ values were 30.8 and 32.0, respectively (range 15–37). The correlation between upE and ORF1a C_t_ values was high (r = 0.94, p<0.001, Spearman’s ρ). The median C_t_ for the upE target from 106 nasopharyngeal swab samples was 32.0; from 8 sputum samples, 24.5; and from 6 bronchoalveolar lavage samples, was 24.5 (p<0.001, Kruskal-Wallis test) (Figure 1). When restricted to hospitalized patients, the median C_t_ values for nasopharyngeal swab, sputum, and bronchoalveolar lavage samples were 31.0, 25.0, and 26.0, respectively (p<0.001, Kruskal-Wallis test).

**Figure 2:**
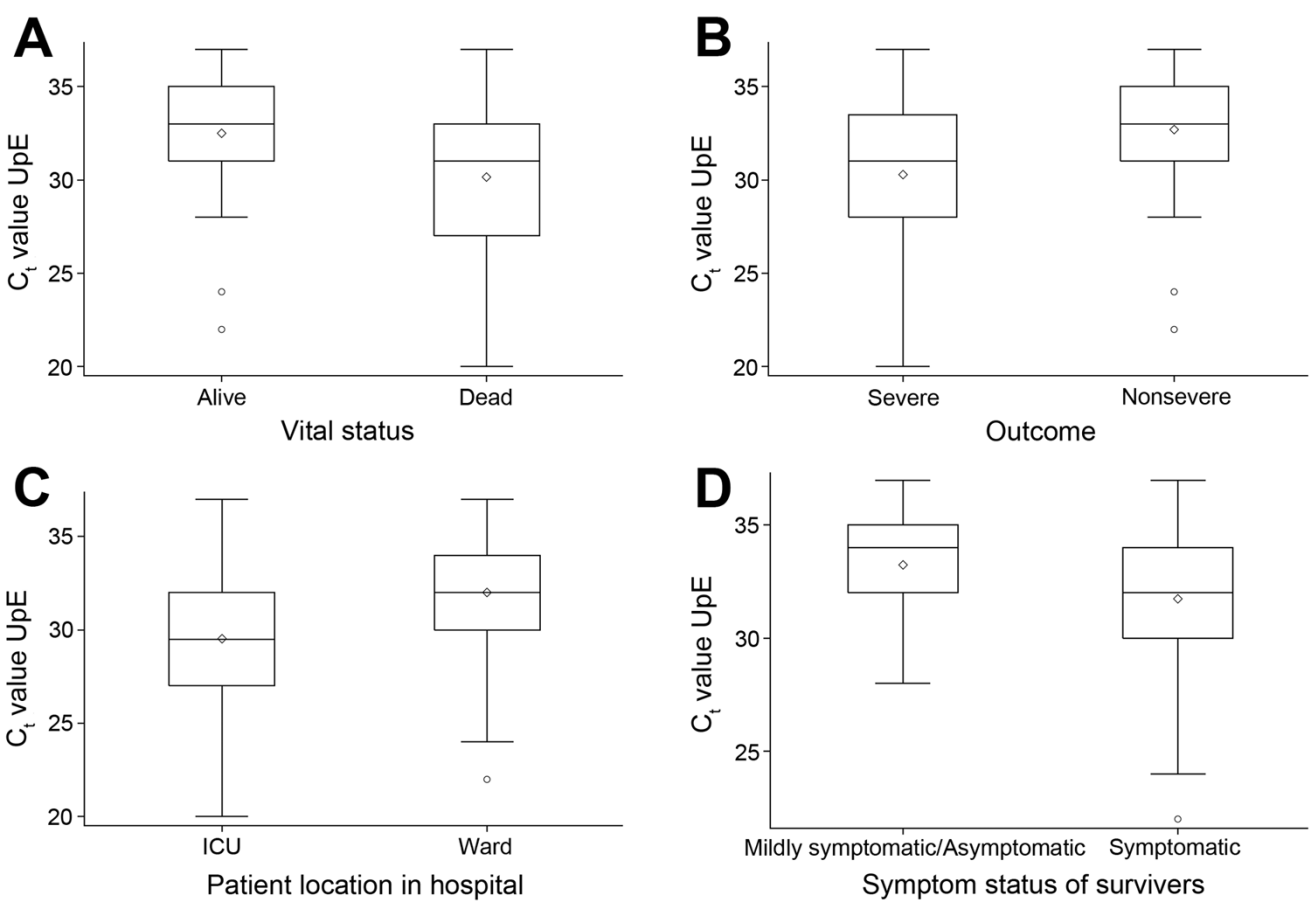
Box plot of C_t_ values for 102 patients infected with MERS-CoV by severity status. Kingdom of Saudi Arabia, 2014. A) Patients who were alive (n = 61) versus dead (n = 41) at the time of follow-up chart review or phone contact. Wilcoxon rank-sum test, p = 0.0087. B) Patients who had a severe outcome (death or ICU admission, n = 48) versus nonsevere outcome (n = 54). Wilcoxon rank-sum test, p = 0.0036. C) Patients who were admitted to the ICU (n = 36) versus the regular ward (n = 31). Wilcoxon rank-sum test, p = 0.014. D) Among patients who survived, symptomatic (n = 30) versus mildly symptomatic/asymptomatic (n = 31). Wilcoxon rank-sum test, p = 0.08. Box and whiskers plot features are as follows: central line in box is median, bottom line of box is first quartile (25%), top line of box is third quartile (75%), diamond is mean, bottom of whiskers is first quarter minus 1.5 × interquartile range, top of whiskers is third quarter plus 1.5 × interquartile range, and dots are outliers. C_t_ , cycle threshold; ICU, intensive care unit; MERS-CoV, Middle East respiratory syndrome coronavirus.

The remaining analysis is restricted to the 102 patients with nasopharyngeal swab samples and known outcome status. Of these 102 patients, 41 (40%) died. The C_t_ values for the upE target for 41 patients who died were significantly lower than those for 61 patients who survived (medians 31.0 and 33.0, respectively, p = 0.009, Wilcoxon rank sum test) (Figure 2, panel A). Using the composite severity variable, we categorized 48 (47%) illnesses as severe; for these patients, Ct values were significantly lower than for the 54 patients without severe disease (medians 31.0 and 33.0, respectively, p<0.0036, Wilcoxon rank sum test) (Figure 2, panel b). Sixty-seven (66%) patients were hospitalized. C_t_ values were significantly lower for the 36 hospitalized patients admitted to the ICU than for the 31 hospitalized patients admitted the general ward (medians 29.5 and 32.0, respectively, p = 0.014, Wilcoxon rank sum test) (Figure 2, panel C). Of the 61 patients who survived, Ct values were not significantly lower for the 30 symptomatic patients than they were for the 31 mildly symptomatic or asymptomatic patients (medians 32.0 and 34.0, respectively, p = 0.08, Wilcoxon rank sum test) (Figure 2, panel D). We found similar associations when we categorized C_t_ values as low, medium, and high (Table 1). The findings were similar for ORF1a (data not shown).

**Table 1:** C_t_ for upE gene based on several indicators of severity of illness for 102 patients infected with MERS-CoV, Kingdom of Saudi Arabia, 2014*

Indicator	Virus load			p value†
	Low, C_t_<26, no. (%)	Medium, C_t_ 27–33, no. (%)	High, C_t_>34, no. (%)	
Vital status				
Died, n = 41	8 (20)	23 (56)	10 (24)	0.0044
Survived, n = 61	2 (3)	32 (52)	27 (44)	
Composite severity status				
Severe,‡ n = 48	8 (17)	28 (58)	12 (25)	0.0060
Not severe, n = 54	2 (4)	27 (50)	25 (46)	
Hospital location§				
ICU, n = 36	8 (22)	21 (58)	7 (19)	0.018
General ward, n = 31	2 (6)	16 (52)	13 (42)	
Symptom status¶				
Symptomatic, n = 30	2 (7)	18 (60)	10 (33)	0.064
Asymptomatic, n = 30	0	14 (47)	16 (53)	

*Ct, cycle threshold; ICU, intensive care unit; MERS-CoV, Middle East respiratory syndrome coronavirus. †Ordinal χ^2^ test with 1 degree of freedom. ‡Among admitted patients. §Severe is a composite of patients who died and patients who were admitted to the ICU.  ¶Among surviving patients.

**Figure 3:**
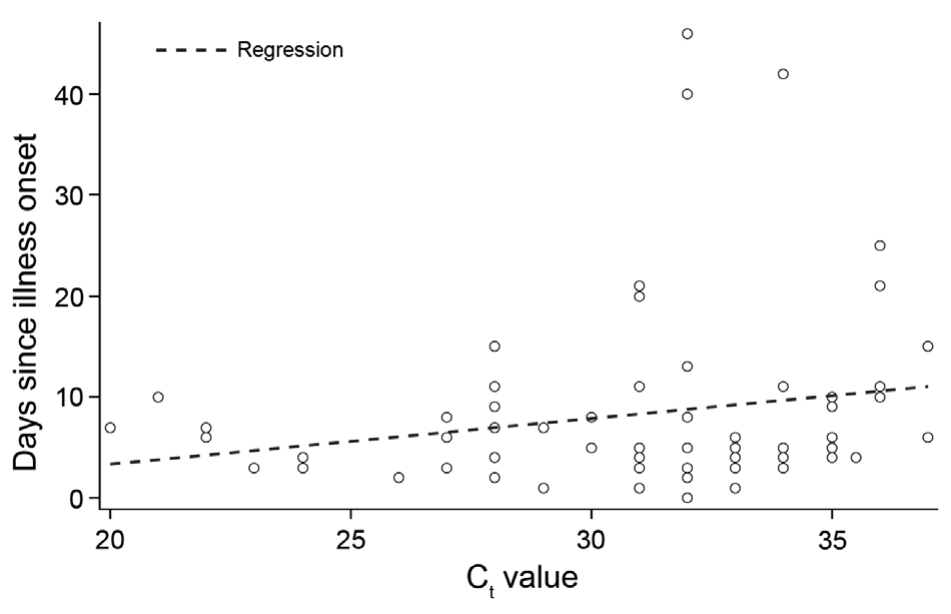
Relationship between days since illness onset and upE C_t_ values for MERS-CoV from nasopharyngeal swabs, Kingdom of Saudi Arabia, 2014. C_t_, cycle threshold; MERS-CoV, Middle East respiratory syndrome coronavirus; upE, upstream of E gene.

Age was not associated with C_t_ when assessed either as a continuous variable (r = −0.07, p = 0.48 Spearman’s ρ) or as quartiles (p = 0.91, Kruskal-Wallis test). Of the 73 patients for whom information was available on days from illness onset to swab sample collection, we found correlation between the days since onset and C_t_ as continuous variables (r = 0.26, p = 0.029, Spearman’s ρ) (Figure 3) with higher virus load found earlier in the course of illness. Among patients for whom the presence (52 patients) or absence (16 patients) of underlying illness was noted, the C_t_ values did not differ significantly (medians 31.5 and 31.0, respectively, p = 0.48, Wilcoxon rank sum test). For only 13 surviving hospitalized patients was enough information available to calculate the length of hospital stay (median 7 days), and C_t_ and length of stay were not correlated for these patients (r = −0.02, p = 0.94, Spearman’s ρ). C_t_ values did not differ significantly by hospital in which the sample was collected (p = 0.33, Kruskal-Wallis test).

In univariate logistic regression, increased probability of death was predicted by older age, presence of underlying illness, and lower C_t_ (Table 2). In multivariable analysis, being >60 years of age (odds ratio [OR] 11.7, 95% CI 2.00–67.9) and having an underlying illness (OR 5.19, 95% CI 1.08–25) were the strongest predictors of death. Lower C_t_ values remained predictive of death when adjusted for age and presence of underlying illness; the odds of death increased 17% for each 1 point drop in C_t_ (OR 1.17, 95% CI 1.01–1.35). For the composite severe outcome, the same factors were identified in univariate and multivariate analysis; C_t_ was borderline significant (OR 1.16, 95% CI 1.00–1.34) when adjusted for age and underlying illness (Table 2). We found similar regression results with ORF1a– when we adjusted for underlying illness and age with a positive association between lower ORF1a C_t_ values and death (OR 1.21, 95% CI 1.04–1.40) and severe disease (OR 1.19, 95% CI 1.01–1.40).

**Table 2:** Results of logistic regression for risk of death and severe outcome (death or ICU admission) for 102 patients with MERS-CoV infection, Kingdom of Saudi Arabia, 2014*

Outcome	Died, n = 41, vs. survived, n = 61			Severe, n = 48, vs. not severe, n = 54	
	Univariate OR (95% CI)	Multivariable OR (95% CI)		Univariate OR (95% CI)	Multivariable OR (95% CI)
Virus load, 1 point decrease in C_t_	1.18 (1.06–1.33)	1.17 (1.01–1.35)		1.20 (1.06–1.35)	1.16 (1.00–1.34)
Age, y, grouped in quartiles					
8–28, n = 23	Referent	Referent		Referent	Referent
29–47, n = 21	2.67 (0.57–12.4)	2.79 (0.49–15.8)		4.10 (0.92–18.4)	5.21 (0.88–30.9)
48–60, n = 27	5.33 (1.28–22.3)	3.42 (0.68–17.1)		8.33 (2.00–34.9)	5.13 (0.98–27.0)
>60, n = 19	18.7 (3.82–91.2)	11.7 (2.00–67.9)		25.0 (4.85–129)	14.0 (2.19–89.7)
Age missing, n = 12	6.67 (1.27–35.0)	7.19 (1.17–44.1)		9.33 (1.76–49.6)	12.5 (1.80–86.3)
Male sex, n = 76	2.20 (0.83–5.84)	NA		2.50 (0.97–6.44)	NA
Underlying illness					
No, n = 16	Referent	Referent		Referent	Referent
Yes, n = 52	6.93 (1.76–27.4)	5.19 (1.08–25.0)		8.14 (2.25–29.5)	7.12 (1.55–32.7)
Unknown, n = 34	0.93 (0.20–4.3)	1.43 (0.25–8.29)		0.64 (0.15–2.70)	0.97 (0.18–5.21)
Week of specimen collection					
March 26–April 7, n = 12	Referent	NA		Referent	NA
April 8–21, n = 45	0.57 (0.16–2.07)	NA		0.44 (0.12–1.66)	NA
April 22–May 5, n = 36	0.27 (0.07–1.07)	NA		0.32 (0.08–1.26)	NA
May 6–16, n = 9	0.57 (0.10–3.3)	NA		0.63 (0.11–3.7)	NA

*ORs for C_t_ for upE target are the odds of having outcome for each 1 point decrease in C_t_. C_t_, cycle threshold; ICU, intensive care unit; MERS-CoV, Middle East respiratory syndrome coronavirus; NA, not included in multivariate model; OR, odds ratio.

## Discussion

We found an association between higher virus load of MERS-CoV detected in the upper respiratory tract, as indicated by lower C_t_ values, and worse clinical outcome, including death and admission to the ICU. This finding is derived from a large number of MERS-CoV patients from KSA during the upsurge in cases during spring 2014, when MERS-CoV was diagnosed in patients with a spectrum of clinical illness (*17*). Our findings did not change when we adjusted for other risk factors for severe outcome and potential confounders. Although the exact pathophysiology of MERS-CoV infection in the lung is still unknown, more virions could lead to worse lung damage either by direct destruction of respiratory epithelial cells or by triggering a more vigorous inflammatory response (*21*). In severe acute respiratory syndrome coronavirus infections, a similar association was observed between worse outcome and higher virus load, as measured in nasopharyngeal, serum, and fecal samples (*22*). Why some patients have higher MERS-CoV virus load is unclear. Virus load might be related to the inoculum size at the time of infection; anecdotally, primary MERS-CoV infections, which are probably more likely to be acquired from environmental exposure (e.g., camels) than from person-to-person spread, tend to have worse outcomes, even among younger patients, although this factor could reflect other risks or case ascertainment bias (*23*,*24*). Virus load might also be related to host factors, such as the immune response to the virus, which in turn could be affected by intrinsic factors, such as the presence of underlying illness or host genetics.

This association between outcome and virus load in the upper respiratory tract has been demonstrated previously for other viral infections. Children with higher respiratory syncytial virus loads are more likely to be hospitalized and require mechanical ventilation (*10*,*12*,*15*). Some studies have found higher virus load for influenza viruses among patients who were sicker (*14*,*25*). Higher virus loads of human bocavirus in nasopharyngeal aspirate samples were associated with greater severity of illness among Chinese children (*16*). Other studies, however, have not shown an association between virus load and illness severity (*11*–*13*).

Clearly the specimen type can affect virus load. A previous study from KSA showed that median C_t_ values were lower for MERS-CoV from tracheal aspirate and bronchoaveolar lavage samples than for nasopharyngeal swab samples, presumably reflecting higher virus load in the lower respiratory tract (*20*). Moreover, lower respiratory tract samples, including expectorated sputum, are positive for MERS-CoV when upper respiratory tract samples were negative (*26*,*27*). We observed similar findings with bronchoalveolar lavage and sputum yielding higher virus load than nasopharyngeal specimens, although this could have reflected that these specimens were only available from sicker patients or been due to chance with so few bronchoalveolar or sputum specimens.

Because the severity of patients most likely was associated with the available specimen (e.g., only intubated patients have bronchoalveolar lavage), we restricted our analysis to patients with nasopharyngeal swab samples, which is the recommended specimen for initial MERS-CoV diagnosis according to KSA MOH guidelines (*18*). The clinical implication of measuring virus load in upper respiratory tract specimens for MERS-CoV is not clear because the virus load probably reflects virus replication in the nasopharyngeal epithelial cells. We assumed that virus load in the upper respiratory tract correlated with that in the lung, although this assumption is not known definitively. By limiting the analysis to virus load among bronchoalveolar lavage specimens, we would have been able to more directly compare virus load in the lung with clinical severity; however, too few of these specimens were available to conduct this analysis.

We showed that several other risk factors were associated with death and severe outcome on multivariable analysis. The strongest risk factors were being elderly and having an underlying illness. Although several studies have shown underlying illness to be common among MERS-CoV patients, including those who died, few have quantified the risk for underlying illness in severe outcomes (*5*,*8*,*28*). We showed that the presence of underlying illness elevated the odds of severe outcome or death by 7–8-fold. The higher risk for severe outcomes earlier in the course of the Jeddah outbreak probably was due to the increase in contact tracing as the outbreak progressed in Jeddah, leading to detection of a greater spectrum of clinical MERS-CoV disease later in the outbreak.

Our analysis is subject to several possible limitations. C_t_ values are a semiquantitative measure of virus load and therefore only reflect relative virus loads. Without reference to a known standard curve for each real-time RT-PCR run, we were not able to assign specific virus load thresholds to severity categories. Run-to-run variability could have existed in C_t_–virus load relationships, although the extraction method, real-time RT-PCR, and equipment remained the same throughout this period in the Jeddah Regional Laboratory. If real-time RT-PCR is 100% efficient, a 1-unit change in C_t_ indicates an ≈2-fold difference in concentration of virus copies, although such conditions are rarely met in real-world practice (*12*). C_t_ values have been used in other reports as an indicator of MERS-CoV virus load (*20*). Second, specimens were collected from multiple hospitals and the home setting for asymptomatic contacts. This could have led to variability in quality of the specimens, and/or handling and shipping practices in getting specimens to the Jeddah Regional Laboratory. However, unless systematic differences were present by site in terms of the severity of illness affecting specimen collection, this variability should have been nondifferential and biased the findings toward the null. Moreover, we observed no difference in the median C_t_ across hospitals. Third, data for other outcomes indicative of severity of illness were either missing in some patients (e.g., days since onset, length of hospital stay) or not available at all in the current dataset (e.g., mechanical ventilation, oxygen requirements). Fourth, some patients initially identified by the MOH as asymptomatic and not hospitalized did not have medical records for review, and data were collected by phone for these patients; some of these patients were unable to be contacted and so we could not verify the initial data from the MOH, leading to potential misclassification for some variables. Finally, without systematically sampling and testing sequential specimens in the same patient during the course of illness, we are unable to determine the temporal sequence in the association between virus load and outcome, i.e., we cannot determine whether higher virus load causally leads to severe outcomes

Although we present evidence for an epidemiologic association between virus load and severity of MERS-CoV illness, the clinical implications of our findings are unclear. At this time, MERS-CoV has no known treatment, other than supportive care. Several approaches to treatment, including antiviral drugs and immunotherapy are being investigated (*29*). More data are needed on whether modulation of virus load by therapeutic agents can affect clinical outcomes.
